# Identifying Hidden Viable Bacterial Taxa in Tropical Forest Soils Using Amplicon Sequencing of Enrichment Cultures

**DOI:** 10.3390/biology10070569

**Published:** 2021-06-22

**Authors:** Chakriya Sansupa, Sara Fareed Mohamed Wahdan, Terd Disayathanoowat, Witoon Purahong

**Affiliations:** 1 Department of Biology, Faculty of Science, Chiang Mai University, Chiang Mai 50200, Thailand; chakriya_s@cmu.ac.th; 2 Department of Soil Ecology, UFZ-Helmholtz Centre for Environmental Research, 06120 Halle (Saale), Germany; sara-fareed-mohamed.wahdan@ufz.de; 3 Botany Department, Faculty of Science, Suez Canal University, Ismailia 41522, Egypt; 4 Research Center in Bioresources for Agriculture, Industry and Medicine, Chiang Mai University, Chiang Mai 50200, Thailand; 5 Research Center of Microbial Diversity and Sustainable Utilization, Chiang Mai University, Chiang Mai 50200, Thailand

**Keywords:** eDNA, enrichment culture, amplicon sequencing, soil bacteria, bacterial community, culture-dependent, culture-independent, bacterial function

## Abstract

**Simple Summary:**

The study of a microbial community nowadays mostly relies on environmental DNA (eDNA)-based amplicon sequencing. However, some studies report that this method is not able to capture all bacterial taxa in the community. This study presents an enrichment culture-based amplicon sequencing method to estimate the proportion of culturable bacteria in soil. A bacterial community derived from this method was compared with those derived from culture-independent methods (eDNA-based amplicon sequencing). This study revealed that the majority of cultured bacteria were rare or completely absent in the community detected by the culture-independent method. Nevertheless, the dominant bacterial Operational Taxonomic Units (OTUs) were also observed, as 8 out of the 30 most frequently detected bacteria from eDNA were found in the enrichment cultures. The method proposed in this study could extend bacterial community’s information derived from the culture-independent method. Furthermore, the enrichment culture-based amplicon sequencing method could be a promising tool for quick screening of a culturable bacterial community and its associated function for various applications.

**Abstract:**

This study aims to estimate the proportion and diversity of soil bacteria derived from eDNA-based and culture-based methods. Specifically, we used Illumina Miseq to sequence and characterize the bacterial communities from (i) DNA extracted directly from forest soil and (ii) DNA extracted from a mixture of bacterial colonies obtained by enrichment cultures on agar plates of the same forest soil samples. The amplicon sequencing of enrichment cultures allowed us to rapidly screen a culturable community in an environmental sample. In comparison with an eDNA community (based on a 97% sequence similarity threshold), the fact that enrichment cultures could capture both rare and abundant bacterial taxa in forest soil samples was demonstrated. Enrichment culture and eDNA communities shared 2% of OTUs detected in total community, whereas 88% of enrichment cultures community (15% of total community) could not be detected by eDNA. The enrichment culture-based methods observed 17% of the bacteria in total community. FAPROTAX functional prediction showed that the rare and unique taxa, which were detected with the enrichment cultures, have potential to perform important functions in soil systems. We suggest that enrichment culture-based amplicon sequencing could be a beneficial approach to evaluate a cultured bacterial community. Combining this approach together with the eDNA method could provide more comprehensive information of a bacterial community. We expected that more unique cultured taxa could be detected if further studies used both selective and non-selective culture media to enrich bacteria at the first step.

## 1. Introduction

Microbes play important roles in all ecosystems [1]. Many of them have been considered as vital drivers in bio-geological processes [2]. Certain microbes are found to be beneficial in a variety of ways, such as enhancing plant production [3] or controlling pathogens [4]. However, they also possess some adverse effects, such as causing plant diseases [5,6]. Information about microbial species living in an ecosystem of interest at different points in time may lead to a better understanding of ecosystem dynamics, functions and health. 

Microbial diversity, community composition and their dynamics have been of significant scientific interest for decades. The traditional culture-based method is a primary approach to investigate culturable microorganisms in microbiological studies. The approach provides a living part of the community and could contribute microbial material that can be used in subsequent researches [7,8]. However, it appears that only a tiny part of the microbial community (e.g., 0.1% to 1.0% of soil bacteria) can be cultured [9]. Consequently, recent microbial community studies have focused on an eDNA-based community, which could provide an overview of microbial structure in an individual environment. A vast number of microbes can be detected by amplicon sequencing of the 16S rRNA gene using high-throughput technologies. However, several studies found that the eDNA failed to detect certain cultured taxa in a community [10,11]. This problem is found to be related to the fact that the quantities of those bacterial taxa are far below the detection limit of the current sequencing technologies (10 cells/mL for Illumina sequencing) as well as the PCR and primer biases [12]. Recent studies have stated that the paradigm that states that only 1% of microbes are culturable should be discarded, as the number of cultured bacteria is gradually increased by several modified methods [13,14]. Therefore, it is encouraging to combine both culture-based and eDNA-based methods to better represent the picture of the microbial community. 

Forest soils are one of the most complex systems that contain multiple habitats and a broad range of microbial diversity [15]. Bacteria in forest soil are the main actors and facilitators in many biogeochemical processes [15]. It is well known that bacteria are important drivers for nitrogen fixation in soil [16]. Furthermore, they are also a part of several processes in the nitrogen cycle including nitrification, denitrification and ammonification [17,18]. Besides, forest soil bacteria are considered as crucial decomposers degrading organic material (cellulose, hemicellulose and pectin, for example) [19,20,21] and solubilizing soil macro- and micronutrients (such as phosphorus, potassium and sulfur) [15,22,23]. Moreover, they can also act as a plant pathogen or as a supporter for plant disease resistance [24]. 

Bacteria, both viable and dead, can be found in forest soils. The viable part can be divided into three forms, which are active, potentially active and dormant [8]. All these forms play important roles in ecosystem functions. The active bacterial form normally performs a function at a current time, whereas the other two forms can play roles on soil ecosystem functions after environmental changes [8,25]. Other important groups of bacteria that can be of particular interest in forest soil are rare taxa, as they have the ability to perform a variety of soil functions [26]. Rare taxa are those with a low abundance (<0.1% relative abundance) detected by eDNA [27]. According to a recent study, rare taxa could be significant drivers of multiple functions other than the abundant ones [26]. Furthermore, they can also serve as microbial seed banks, which are vital facilitators of many ecosystem functions after disturbance [27,28]. To identify such rare bacterial taxa is difficult based on information from eDNA sequencing alone as it is impossible to distinguish between rare taxa of dead and dormant cells. This can be achieved by integrating the eDNA sequencing approaches with culturing techniques [29].

This present study aims to estimate bacterial diversity and community composition detected in forest soil, using both culture-based and eDNA-based methods. However, the traditional time-consuming culture method was modified by replacing colony-picking and culture purification steps with amplicon sequencing. The combination of culture enrichment and amplicon sequencing methods allows the quick screening of the culturable bacterial community. Four main questions were addressed towards the soil’s bacterial richness and community composition derived from enrichment cultures and eDNA: (i) do culture-based method contributes unique bacterial taxa that could not be detected by eDNA?; (ii) how many unique and shared bacterial taxa detected by culture-based and eDNA-based methods?; (iii) what and how many potential functions are captured with culture-based method as compared with the eDNA and iv) which parts (abundant vs rare taxa) of bacterial community are detected by culture-based method? The study was carried out using forest soil samples and amplicon sequencing of enrichment cultures derived from three non-selective media including nutrient agar (NA), plate count agar (PCA) and tryptone soy agar (TSA). We hypothesized that both dominant and rare bacterial taxa from diverse functional groups can be detected using the amplicon sequencing of enrichment cultures. Fast growing bacteria are expected to dominate in datasets of the amplicon sequencing of enrichment cultures as compared with eDNA.

## 2. Materials and Methods

### 2.1. Soil Collection

This study was conducted in bamboo-deciduous forest in, northern Thailand (18°32′23′′ N, 99°34′47″ E). The forest was dominated by *Dendrocalamus membranaceous* and *Bambusa bambos.* Soil samples were collected in June 2018. Soil texture was classified as clay (77% of clay, 13% silt and 10% sand) containing main physicochemical properties as follows: pH (H_2_O) 7.19, 23.34% moisture, 6.65% soil organic matter, 0.33% total nitrogen (N), 126.01 mg/kg Phosphorus (P) and 326.66 mg/kg Potassium (K). A total of six soil samples were collected (*n* = 6). In detail, six replicated plots (5 × 5 m) at least 20 m apart were first set up in the forest. Five subsamples were taken to a depth of 10 cm from each plot using soil augur 10 cm in diameter. The subsamples were pooled into one composite sample, then homogenized and filtered through a 2 mm sieve. Subsequently, each composite soil sample was separated into two subsamples, one stored at 4 °C for enrichment culture-based method and the other stored at −20 °C for eDNA-based study. 

### 2.2. Bacterial Enrichment

**Figure 1:**
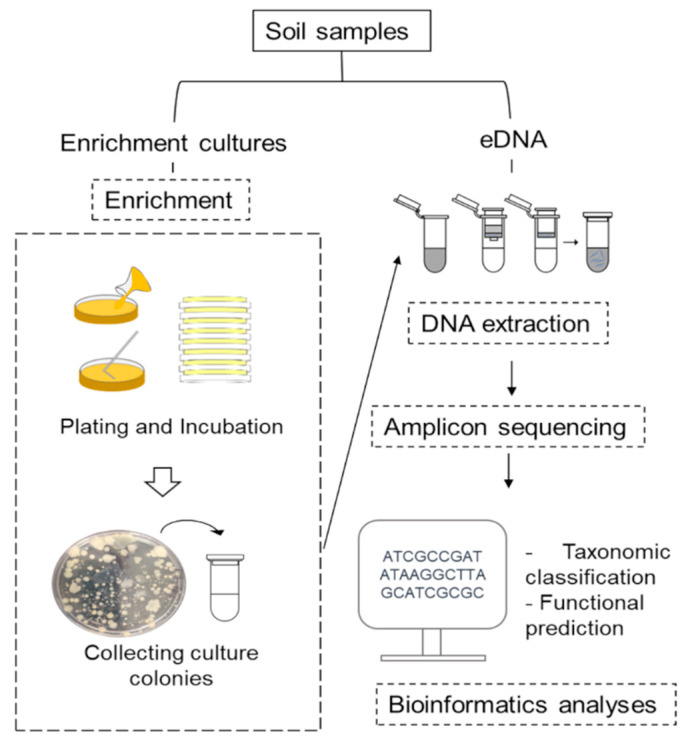
Workflow of amplicon sequencing based on enrichment culture vs eDNA.

Soil bacteria were cultured within 24 h after soil collection. For cultivation of soil bacteria, three non-selective media, including nutrient agar (NA), plate count agar (PCA) and tryptone soy agar (TSA), were used as a model to observe a cultured bacterial community in the soil. Since these media enrich a broad range of bacteria species, several different bacterial species could be detected. In detail, 1 gram of soil was added into 9 mL sterilized 0.85% NaCl and vortexed thoroughly to homogenize soil suspension. A 100 μL of soil suspension was subsequently added to culture media by two culture methods: (i) the spread plate method and (ii) the pour plate method. Whilst the former method was used to capture strictly aerobic and facultative anaerobic bacteria, the latter was expected to capture certain bacterial taxa that required less oxygen or certain anaerobic bacteria. It may be possible to capture more living bacteria in the samples by using both methods. Subsequently, the cultured plates were incubated under aerobic conditions by inverting the plate and placing it in an oxygen presented atmosphere, as well as anaerobic conditions by inverting the plates and placing them in an anaerobic jar. All plates were incubated at 25 °C for 72 h. After that, all colonies that immersed in/on each culture media were collected into collection tubes. Each collection tube held a mixture of bacterial colonies from the spread plate and pour plate methods. Overall, 18 collection tubes were acquired (one for each sample; NA = 6, PCA = 6, and TSA = 6). The colony mixtures were stored at −20 °C until further analyses (Figure 1). 

### 2.3. DNA Extraction, Amplicon Sequencing and Sequence Processing

Genomic DNA was extracted from 300 µL of the colony suspension collected from each medium (NA, PCA, TSA) and 0.25 g of soil, using a NucleoSpin^®^ Soil DNA extraction kit, according to the manufacturer’s instructions. DNA samples were subsequently amplified using bacterial primer pair Bact341F (5′CCTACGGGNGGCWGCAG-3′) and Bact785R (5′-GACTACHVGGGTATCTAATCC-3’) [30] which captured V3-V4 region of bacterial DNA. The amplicons were sequenced using an Illumina MiSeq (Illumina, Inc., San Diego, CA, USA) 2 × 300 bp paired-end strategy, according to the manufacturer’s manual. All amplification and sequencing steps were performed at RTL Genomics (Lubbock, TX, USA). Raw sequence datasets derived from the Illumina analysis are available in the National Center for Biotechnology Information (NCBI) under BioProject accession number PRJNA712947. 

Subsequently, raw sequences were analyzed using Mothur 1.33.3 [31]. The raw sequences, with ≥20 bp overlap, were combined to generate paired-end sequences. The paired sequences were filtered to get high quality sequences with the following characteristics: sequence length between 390–520 bp, Phred score ≥ 25, and maximum length of 20 homopolymers in the sequence and without ambiguous nucleotides. Chimeric sequences were removed using UCHIME 4.2.40 [32] as implemented in the Mothur. Operational Taxonomic Units (OTUs) were generated by clustering together the sequences with ≥97% identity. A representative sequence was randomly chosen from each OTU and the bacterial taxonomy was determined with the SILVA v132 sequences database [33]. Bacterial functions were assigned to each OTU, based on their taxonomy, using functional prediction software called FAPROTAX [34]. The FAPROTAX predicted the ecological relevant function of bacteria based on the literature of culturable taxa. A taxon was predicted to a specific function when all culture representative of that taxon performs the function. This present study used the latest version of the FAPROTAX database (version 1.2.4) and an additional database of soil bacteria presented in Sansupa et al. [35] as a key to predict bacterial functions. The prediction was performed according to FAPROTAX instructions available on the FAPROTAX web page (http://www.loucalab.com/archive/FAPROTAX/lib/php/index.php?section=Instructions accessed on 25 January 2021). After that, OTUs containing 1–3 sequences (singletons, doubletons and tripletons), which could potentially originate from sequencing error, were removed from the datasets. Lastly, the datasets were normalized to the smallest number of sequence reads per sample (7724 sequence reads/ sample) using “rrarefy” function from Vegan package [36] in R programing [37]. These normalized datasets were used for further statistical analyses. To verify the actual rare bacterial taxa in eDNA-based community, we additionally normalized bacterial datasets using different sequencing depths, including 9000 and 10,000 sequence reads/samples. Two and four datasets contained less than 9000 and 10,000 sequence reads were excluded. Bacterial OTUs with average relative abundance <0.1% in all analyzed sequencing depths were defined as rare OTUs [27]. 

### 2.4. Statistical Analyses

Statistical analyses were performed in R programing [37] and PAST program version 2.17c [38]. Differences between community composition of bacteria derived from culture-based (NA, PCA, and TSA) and eDNA-based (eDNA) methods were tested using nonparametric multivariate analysis of variance (NPMANOVA). Non-metric multidimensional scaling (NMDS) was performed to identify and visualize bacterial distribution patterns across four sample groups. The NPMANOVA and NMDS were calculated based on two dissimilarity matrices, including Bray–Curtis for abundance data and Jaccard for presence/absence data (data excluded the effected of read count in each OTU; detected OTUs in each sample were considered as “presence” (1), whilst undetected OTUs were considered as “absences” (0)). Furthermore, correlation between bacterial OTUs derived from cultured samples and those derived from eDNA samples was tested using Spearman’s rank method. Moreover, the Spearman’s rank correlation method was used to test the correlation of bacterial OTUs among cultured samples (NA, PCA and TSA). Differences in the relative abundance of bacterial taxa obtained from enrichment cultures and eDNA were tested using Wilcoxon rank-sum tests and visualized on a heat tree that was created by the Metacoder package [39] in R programing. Furthermore, differences in functional redundancy (the number of bacterial OTUs that potentially perform same functions) of each predicted functions were tested, using t-tests, and visualized on STAMP software [40].

## 3. Results

### 3.1. General Overview of Bioinformatics and Taxonomic Information of Becteria Detected by Culture-Based and eDNA-Based Methods

**Figure 2:**
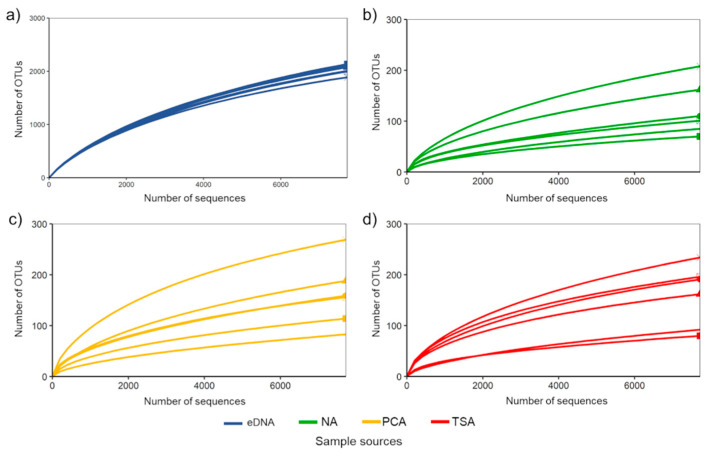
Rarefaction curves of observed bacterial OTUs recovered from (**a**) eDNA and enrichment cultures based on (**b**) nutrient agar (NA), (**c**) plate count agar (PCA) and (**d**) tryptone soy agar (TSA).

A total of 482,840 high-quality and abundant (no singletons, doubletons and tripletons) sequence reads, presenting 3913 OTUs, were derived from this study. However, after normalization, 3894 OTUs remained and were used for further analyses. Rarefaction curves of bacterial OTUs derived from both eDNA and culture samples gradually reached saturation at the analyzed sequencing depth (7724 reads per sample), indicating that the detected OTUs were sufficient to represent the bacterial richness and community composition in each sample (Figure 2). Furthermore, we showed that increasing sequencing depth had no significant effect on the number of rare OTUs in this study (Table S1). Specifically, almost all rare bacterial OTUs based on the sequencing depth at 7724 reads per sample were also rare when other sequencing depths (9000 and 10,000 reads per sample) were used.

**Figure 3:**
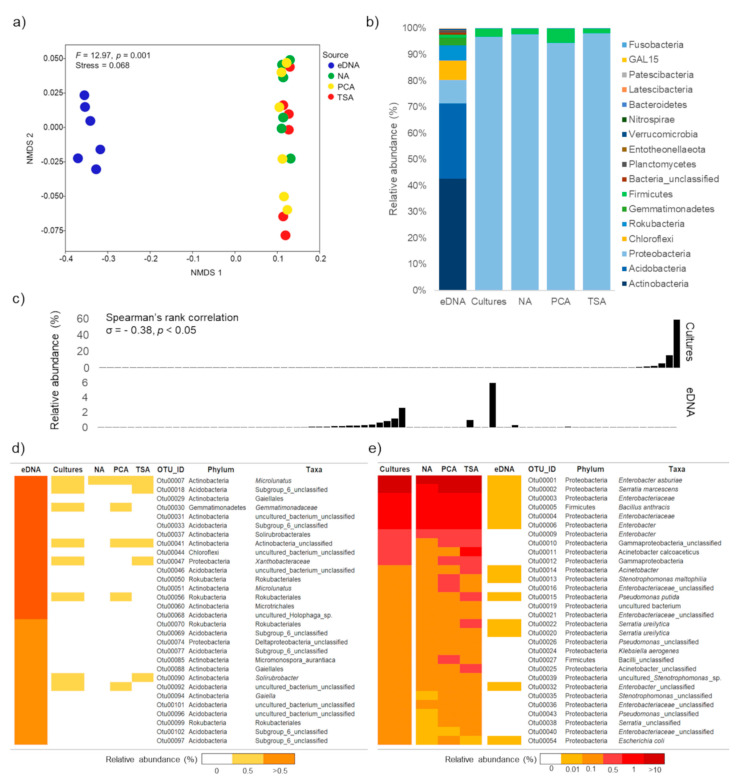
Taxonomic distribution of soil bacteria detected by eDNA- and culture-based amplicon sequencing. (**a**) Non-metric multidimensional scaling (NMDS) ordination of bacterial communities based on Bray-Curtis distance measure, under four different treatments. (**b**) Proportion of bacterial phyla detected in each treatment. (**c**) Spearman’s rank correlation between relative abundance of shared OTUs derived from eDNA and enrichment cultures. Bar plots show relative abundance of shared OTUs. A plot at the same position (x-axis) represents an identical OTU. Plots on baseline (nearly zero) represent OTUs with relative abundance lower than 0.05%. (**d**) Heat map of most abundance OTUs (top 30) based on eDNA method and (**e**) enrichment cultures. Color indicates relative abundance. Darker red represents high relative abundance and white represents non-detected OTUs. Column represents method and type of culture medium. The abbreviations for indicated treatments were as follows: eDNA = eDNA-based methods, cultures = enrichment culture-based method, which is a result from three media: NA = nutrient agar; PCA = plate count agar; and TSA = tryptone soy agar.

The community compositions of bacteria, based on abundance and presence/absence data, derived from the three culture media were different from those derived from eDNA (Figure 3a). Bacterial OTUs in eDNA community was much more diverse than that of culture communities (OTUs richness: eDNA = 2035 ± 37, NA = 123 ± 21, PCA = 162 ± 26 and TSA = 159 ± 25). In total, 16 phyla were found in this study. Whilst 8 out of 16 phyla, including Acidobacteria, Actinobacteria, Bacteroidetes, Chloroflexi, Firmicutes, Gemmatimonadetes, Proteobacteria and Rokubacteria, were detected by both methods, the others, belonging to Entotheonellaeota, Fusobacteria, GAL15, Latescibacteria, Nitrospirae, Patescibacteria, Planctomycetes and Verrucomicrobia, were found specifically in eDNA samples. The most abundant phyla detected by eDNA were Actinobacteria (43%), Acidobacteria (29%) and Proteobacteria (9%), whereas those detected by enrichment cultures were Proteobacteria (96.7%; most of which belonged to Gammaproteobacteria) and Firmicutes (3%) (Figure 3b). Although the taxonomic distribution of bacteria based on abundance and presence/absence data presented similar trends, the proportions of detected phyla shown in presence/absence data were decreased in relation to abundance data. Whilst Actinobacteria (38%), Acidobacteria (31%), Proteobacteria (11%) and Chloroflexi (8%) were found to be dominant in eDNA community, culture community was dominated by Proteobacteria (88%), Actinobacteria (6%) and Firmicutes (3%).

### 3.2. Total Soil Bacterial Community: Unique, Overlap and Prevalent Taxa Derived from Each Method

**Figure 4:**
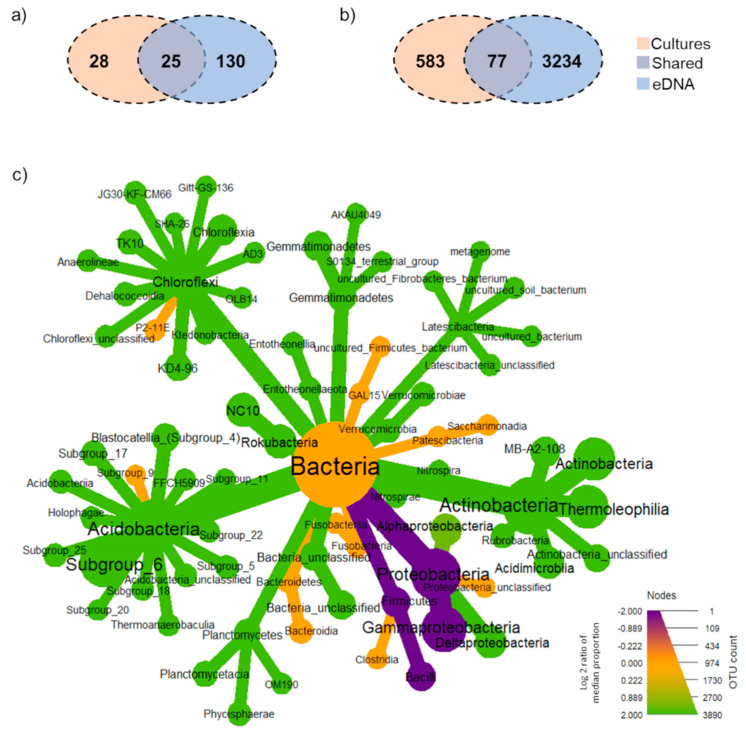
Taxonomic distribution of the total community. The Venn diagram presents the number of shared and unique (**a**) genus and (**b**) OTUs between culture-based and eDNA-based amplicon sequencing approaches. (**c**) Differential heat tree shows bacterial taxa with significant different abundance in total community at phylum and class levels. Node size represents number of observed OTUs. Taxa in purple are significantly higher in the culture community and those in green are significantly higher in the eDNA community. Yellow taxa indicate no difference.

At genus levels, 183 classified genera in total were found, of which 155 and 53 of those were detected by eDNA and culture enrichment, respectively (Figure 4a). Whilst most of the classified genera (*Micromonospora, Rubrobacter, Pseudonocardia* and *Acidothermus,* for example) were found to be unique in eDNA samples, 25 genera (such as, *Klebsiella, Cronobacter* and *Lysinibacillus*) were detected only by culture enrichment. However, approximately 15% of all classified genera, (such as, *Enterobacter, Bacillus, Acinetobacter* and *Streptomyces*) were detected by both methods. 

Considering all OTUs detected in this study as “total community” (cultures + eDNA), 17% of total bacterial communities were detected by a culture-based method using three non-selective media, whereas 85% were detected by eDNA-based method (Figure 4b). In total, 2% of bacteria were detected by both methods. Wilcoxon tests indicated that most bacterial taxonomic groups showed significant differences in their abundances between culture-based and eDNA-based methods. Whilst members of Gammaproteobacteria and Bacilli were significantly higher in the enrichment cultures community, those belonged to Acidobacteria, Actinobacteria and Chloroflexi were prevalent in the eDNA community (Figure 4c). Several rare taxa were found in enrichment cultures, such as *Burkholderiaceae*, *Nitrosomonadaceae, Bacillus* spp., *Cupriavidus*, *Enterobacter asburiae, Serratia* spp. Approximately 88% of all cultured OTUs, including, but were not limited to, *Achromobacter xylosoxidans, Acinetobacter baumannii, Acinetobacter calcoaceticus, Aeromonas, Allorhizobium-Neorhizobium-Pararhizobium-Rhizobium, Ensifer, Hafnia-Obesumbacterium, Klebsiella aerogenes* and *Staphylococcus gallinarum,* were only detected by the culture-based method (Table S2). On the other hand, more than 95% of all OTUs detected by eDNA were unique in eDNA communities, such as *Bryobacter*, Candidatus *Solibacter* and *Blastocatella.*

A focus on the top 30 most abundant OTUs detected by each method found that eigth OTUs detected in enrichment cultures community were also observed in the top 30 taxa of the eDNA community. On the other hand, 13 OTUs detected in eDNA community were found in that of the culture community (Figure 3d,e). This showed that abundance taxa detected by one method can be rare or even completely absent when using another method. In fact, weak negative correlation was detected between relative abundances of 77 shared OTUs derived from eDNA and enrichment cultures (σ = −0.38, *p* < 0.05) (Figure 3c). Furthermore, 21 out of those 77 OTUs were confirmed as rare OTUs based on the analysis of three sequencing depth (Table S1).

### 3.3. Bacterial Taxa Derived from Different Non-Selective Media

No significant differences were found in the community composition of bacteria derived from NA, PCA and TSA. (Figure 3a). Significant positive correlations were found between the abundance of OTUs detected from each medium (σ = 0.44–0.51, *p* < 0.05). A majority of cultured OTUs (approximately 36%) was detected in every medium. For example, the bacterial taxa *Enterobacter* (Otu00001)*, Serratia* (Otu00002)*, Enterobacteriaceae* (Otu00003)*, Bacillus* (Otu00005) were all highly detected in all media with relative abundances greater than 1%. Approximately 30% of cultured OTUs were found in two out of three media, such as *Ensifer* (Otu03875; NA and PCA), *Serratia rubidaea* (Otu03187; PCA and TSA) and *Pseudomonas citronellolis* (Otu01012; PCA and TSA). On the other hand, certain OTUs were found uniquely in each culture medium (NA = 35 OTUs (5%), PCA= 97 OTUs (14%) and TSA = 86 OTUs (13%); Figure S1). For example, OTUs belonging to *Staphylococcus gallinarum*, *Bacillus niabensis* and *Clostridium thiosulfatireducens* were unique to NA, PCA and TSA communities, respectively.

### 3.4. Potential Bacterial Functions Captured by Culture-Based Method as Compared to eDNA

**Figure 5:**
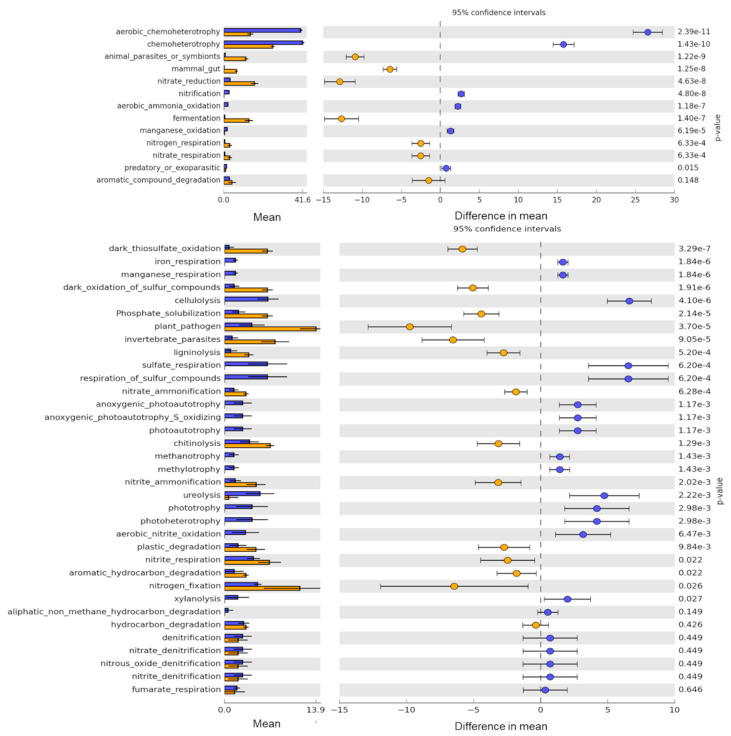
Bacterial associated functions predicted by FAPROTAX. Extended error bar plot indicates significant differences between mean number of bacterial OTUs that potentially performed each function in eDNA (blue) and cultured (orange) samples. Functions overrepresented in the eDNA samples have a positive difference between number of OTUs and functions overrepresented in the culture samples have a negative difference between number of OTUs. Upper section represents functions with average number of OTUs higher than 10 OTUs, whereas the lower section represents functions with those lower than 10 OTUs. *p*-values based on *t*-test are shown at right.

A total of 48 ecologically relevant functions were predicted by FAPROTAX. Diverse detected functions were particularly related to soil ecosystem. These included various biogeochemical cycles which were classified as functions related to (i) C cycle (such as cellulolysis and hydrocarbon degradation), (ii) N cycle (such as nitrogen fixation, nitrification, nitrate ammonification and ureolysis), (iii) P cycle (phosphate solubilization), (iv) microelements and metals (sulfate respiration, manganese oxidation and iron respiration, for example) and (v) general (non-specific) soil functions (such as chemoheterotrophy and fermentation). However, it should be noted that some functions may relate to more than one biogeochemical cycle; chitinolysis is involved with both N and C cycles, for example. This study showed that diverse bacteria detected by the enrichment culture-based amplicon sequencing method have potential to perform diverse soil functions (Figure 5). 

Specifically, 713 OTUs in the total community were assigned to at least one function and 31% (222 OTUs) of those belonged to OTUs that found unique in the enrichment culture community. Although there was high OTU richness in eDNA community, the proportion of functional assigned OTUs was generally low, ranging from 13% to 18%. In contrast, bacterial richness derived from enrichment cultures was low but the proportion of functional assigned OTUs was high (ranged from 33% to 58%). Bacterial OTUs derived from enrichment cultures captured the majority of soil functions that potentially performed by bacteria derived from eDNA. However, there are some functions, such as xylanolysis, methanotrophy, phototrophy and sulfate respiration, that detected only in eDNA community. 

Results of functional redundancy within the bacterial communities revealed that the number of bacterial OTUs capable of certain functions, such as nitrate reduction, nitrogen respiration, dark oxidation of sulfur compounds and fermentation, was significantly higher in enrichment cultures than that in eDNA (*p* < 0.05; Figure 5). Importantly, it was found that although the number of bacterial OTUs capable of ecological functions in enrichment cultures and eDNA community were not significantly different, it appeared that each method contributed different taxonomic groups of bacteria that perform the same functions. For example, *Bradyrhizobium, Methylocapsa* and *Burkholderia* were assigned as nitrogen fixation in eDNA community, whereas in enrichment cultures, *Ensifer* and *Cupriavidus* (Otu01631, Otu01777, Otu01710, Otu01271) were assigned to that function. Another example was nitrous oxide denitrification, which was potentially performed by *Rhodoplanes* in eDNA community and by *Achromobacter* in cultured community. Similar evidence also found in aerobic chemoheterotrophy, aromatic compound degradation, nitrate reduction, and manganese oxidation, for example. All bacterial taxonomic and functional information is presented in Table S3.

## 4. Discussion

### 4.1. Revealed Hidden Viable Bacterial Taxa in Tropical Forest Soils Using Amplicon Sequencing of Enrichment Cultures

Theoretically, eDNA extracted from the soil should provide for all indigenous microorganisms; however, certain taxa might be omitted when using amplicon sequencing [9]. This may be caused by the complexity of soil, which consists of several compounds (e.g. humic acid or salt) that can limit the recovery of microbes during DNA extraction and amplification [41,42]. Moreover, bias of PCR primers and low abundance of DNA could also be an issue [12,43]. However, eDNA-based amplicon sequencing can still be a valuable approach for exploring information on microbial diversity, community composition and their response to natural or human-induced changes e.g., [44,45,46]. Nevertheless, application of enrichment cultures could extend this information.

This study demonstrates that eDNA and enrichment culture-based amplicon sequencing captured different fraction of forest soil bacteria. Differences in community composition and taxonomic distribution implies that specific bacterial groups in total community were revealed by each method. Whilst Actinobacteria, Acidobacteria and Proteobacteria dominated in the eDNA community, supporting previous reports [15,47,48], Proteobacteria and Firmicutes dominated in the enrichment cultures community. Specifically, we present that culture enrichment can provide viable bacterial taxa that are omitted from eDNA. Several taxa, such as *Achromobacter, Acinetobacter* spp. and *Ensifer,* were only observed by the culture method. However, our findings followed the fact that eDNA-based method revealed diverse and high proportions of soil bacteria in a community [9,30]. Consistent with Stefani et al. [10] and Shade et al. [29], we found that both culture-based and eDNA-based provide unique taxa of soil bacteria, approximately 15% and 83% of bacteria in total community were unique in cultures and eDNA community, respectively. 

Furthermore, our study supports the view that cultured taxa omitted from eDNA are likely to represent rare species in an environment [29]. The negative correlation detected between the abundance of OTUs detected by eDNA and enrichment cultures implies that taxa abundant in eDNA are likely to be rare in enrichment cultures and vice versa. Since this study ameliorated the biases during sample analysis by identifying bacterial diversity in both enrichment cultures and eDNA with amplicon sequencing, unique taxa detected by the cultured-base method could be classified as rare taxa (detected less than 0.1% by eDNA). Thus, we suggest that certain rare taxa in soil samples can be identified by an enrichment culture-based amplicon sequencing method. However, this study showed that enrichment culture-based amplicon sequencing not only detected rare taxa but also the abundant ones. Almost one-third of the most abundant OTUs (top 30) detected by eDNA were also detected in enrichment cultures, albeit in low abundance. The observation of viable bacteria, including both rare taxa and dormant taxa extend the information of microbial seed bank which could be important for ecosystem recovery after a disturbance or environmental change [25,49]. Notice should be taken to the fact that the culture method did not out-compete the eDNA in terms of diversity detection, and both methods only revealed different side of the community. Whilst eDNA detected both viable and dead microbes [8,50], enrichment culture only detected the viable ones, some of which were missed by eDNA. Thus, using enrichment cultures along with the eDNA method could present more comprehensive information than using eDNA alone. 

### 4.2. Limited Number of Viable Bacteria Detected by Amplicon Sequencing of Enrichment Cultures and Possible Way to Improve the Method

The result of this study supports the expectation that fast-growing bacteria were likely to be the dominant taxa in culture community. Data based on three non-selective media (NA, PCA and TSA) show that enrichment cultures gathered a large number of Proteobacteria and some Firmicutes, but neglect some other bacterial taxa detected by eDNA, especially those belonging to Acidobacteria and Actinobacteria. A similar trend was also presented by Stefani et al. [10]. The application of resource-rich media in this study provided a favorable condition for fast-growing bacteria [51] which may out-compete slow-growing species and become dominant in the community; thus it was not surprising that many Acidobacteria and Actinobacteria are disappear from the datasets of enrichment cultures. The majority of Acidobacteria were defined as oligotrophs which usually thrive in low nutrient media and grow very slowly [52,53]. Actinobacteria, on the other hand, are also known as slow-growing bacteria [54]; thus, short incubation periods in this study cannot gather many of these taxa. In total, we detected 7 and 33 OTUs from Acidobacteria (*Pyrinomonadaceae* and Acidobacteria_subgroup 6) and Actinobacteria (*Ilumatobacteraceae*, *Geodermatophilus, Agromyces, Microbacterium, Arthrobacter, Glutamicibacter, Paenarthrobacter, Marmoricola, Nocardioides, Microlunatus, Streptomyces* and *Solirubrobacter*). Other taxa that cannot be detected by culture enrichment may also be facing similar issues to those described above. However, a major limitation of culture enrichment was that this method cannot capture unculturable bacteria, which were found to be an important part of the soil bacterial community detected by eDNA. Nevertheless, several studies have suggested some solutions to culture the previously uncultured taxa [55,56]. Modifying culture media and adjusting culture techniques, such as increase incubation times, could provide more diverse taxa in future work. Shade et al. [29] demonstrated that the application of modified rhizosphere isolation media (RIM), which inhibit the growth of *Bacillus mycoides*, can detect more than 10% of viable bacterial OTUs in soil samples. Furthermore, reducing medium pH [52], diluting nitrite broth [57], using modified oligotrophic agar medium [58] and increasing incubation time to 15 days would be recommended in order to increase the number of Actinobacteria and Verrucomicrobia. Besides, using selective media can also increase the proportion of other taxa. For example, applying starch casein agar along with increasing incubation times to 7–14 days [54] may increase the diversity of Actinobacteria. We suggest in further study that various types of media, both selective and non-selective, should be used together to capture diverse bacterial taxa. We believe that more unique culturable bacteria will be detected following the adjustment or modification of the media used in the enrichment culture-based amplicon sequencing method. 

### 4.3. Detection of Diverse Bacterial Functional Groups in Soils by Amplicon Sequencing of Enrichment Cultures

Based on the functional prediction of FAPROTAX software, evidence has been presented here to support the view of Shade et al. [29] that cultured taxa could potentially perform vital soil functions. We found that bacteria detected from enrichment cultures, especially those rare and unique taxa in culture community, may have the potential to provide important functions, including general soil functions and specific soil function related to C, N, P, microelements and metals cycles. For instance, *Ensifer,* usually associated with legume plants, was found to play a role in nitrogen fixation [59]. Both *Micromonospora* and *Rubrobacte* were found to potentially performed nitrate reduction [60,61]; while *Stenotrophomonas and Lysinibacillus* could bring about manganese oxidation [62]; *Acinetobacter* showed potential in aromatic compound degradation [63], respectively. These results show a promising application of the method to screening rare taxa that potentially performed specific functions in soil. As presented by Hobel et al. [64], enrichment culture-based amplicon sequencing could enhance the detection of amylase gene diversity in water samples. However, since this research cultivated bacteria in culture media with materials and conditions that did not precisely reflect the original environment, it is difficult to determine whether the observed bacterial taxa were part of active bacteria or microbial seed banks. In terms of ecosystem dynamics, we support the hypothesis that rare bacterial taxa (including cultured unique taxa) may not affect soil functions in a normal state, but they could be an important driver when the appropriate conditions arises or there are environmental changes [49,65,66]. 

### 4.4. Potential Applications of Enrichment Culture-Based Amplicon Sequencing

Enrichment culture-based amplicon sequencing could be a useful method for detecting the rare and/or viable bacterial taxa that are capable of utilizing specific substrates included in a culture medium. This method can be used for quick screening of viable microbial diversity as well as their associated functions. It is suggested that more comprehensive information on a microbial community can be obtained by using this method along with eDNA. However, it should be noted that while the method could detect live microbes and offer an overview structure of the viable microbial community, it may not directly give an insight overview of novel species. Colony picking and purification, as well as individual colony sequencing, should be included in future research.

Another promising application of this method would be possible on soil samples that contain extremely low microbial abundance or high inhibitors. Sansupa et al. [43] showed that this method can be used to identify viable bacteria that survived in extreme soil conditions in mining areas where microbial DNA cannot be efficiently extracted. Based on these results, some potential soil bacteria are suggested for use as materials for restoration/rehabilitation of highly degraded soil in a limestone mine area. Moreover, the enrichment culture-based amplicon sequencing can be applied to analyze viable fungal community by adapting the culture media to be specific for fungi.

### 4.5. Future Perspective on the Study of Viable Soil Microorganism and Outlook

Cultivation efforts play a significant role in activating dormant microorganisms. For each media, only a subset of viable bacteria will be able to grow and be detected. However, it should be noted that soil microorganisms interact with biotic and abiotic (environmental) factors. In natural soils, ecological interactions play a large role, e.g., viruses, grazing eukaryotes or other prokaryotes may affect the bacterial population dynamics in different ways. The presence of a specific microorganism can drastically alter the population of other organisms, thereby altering the structure and composition of the community and ecosystem [67]. In this current work, we used culture media for bacteria, thus we expect that the bacteria–bacteria interactions are among the most important biotic factors. Future work should also investigate the complex interactions of different organisms in soil systems by modifying enrichment culture-based amplicon sequencing method. For examples, after dormant bacteria are activated during the cultivation, viruses, grazing species and other organisms like fungi maybe also awaken. This opens opportunities to simultaneously study these complex interactions in soils. Although culture-based methods have the potential to activate dormant microbes, some dormant taxa, particularly those belonging to viable but not culturable (VBNC) microbes, may remain hidden as they cannot be cultured using routinely used laboratory media [68]. This might be accomplished, for example, by changing culture medium or employing culture-independent techniques that captured only active microorganisms [69,70].

## 5. Conclusions

Our findings demonstrate that the enrichment culture-based amplicon sequencing method could reveal hidden viable bacterial taxa in soil, detecting both abundant and rare unique viable taxa. Such rare and unique viable taxa can perform a variety of soil functions. Combining this culture-based method with eDNA could provide a better picture of bacterial diversity and community composition in soils. We highlight that the efficiency of the enrichment culture-based amplicon sequencing method is highly dependent on the media selection and culturing conditions. Thus, diverse bacterial taxa are expected to be detected by modifying culture media and conditions.

## Data Availability

Publicly available datasets were analyzed in this study. These data can be found under BioProject accession number: PRJNA712947.

## Supplementary Materials

The following are available online at https://www.mdpi.com/article/10.3390/biology10070569/s1, Figure S1. Venn diagram presents the number of OTUs found in each culture media. The names of unique taxa in each media are presented beside the diagram. The abbreviations indicate media types as follows: NA = nutrient agar, PCA = plate count agar and TSA = tryptone soy agar. Table S1. Rare bacterial OTUs (within a total of 77 bacterial OTUs shared between eDNA and enrichment culture) detected in eDNA sample at different sequencing depths. Table S2. OTUs table shows average relative abundance of all taxa that can be detected by enrichment culture-based amplicon sequencing. Table S3. Bacterial taxonomy and functional information predicted by FAPROTAX.
