# Supplementary figures and images for: Identifying Hidden Viable Bacterial Taxa in Tropical Forest Soils Using Amplicon Sequencing of Enrichment Cultures
Source: Biology (Basel). 2021 Jun 22;10(7):569. doi: 10.3390/biology10070569 (PMC8301126; doi:10.3390/biology10070569)

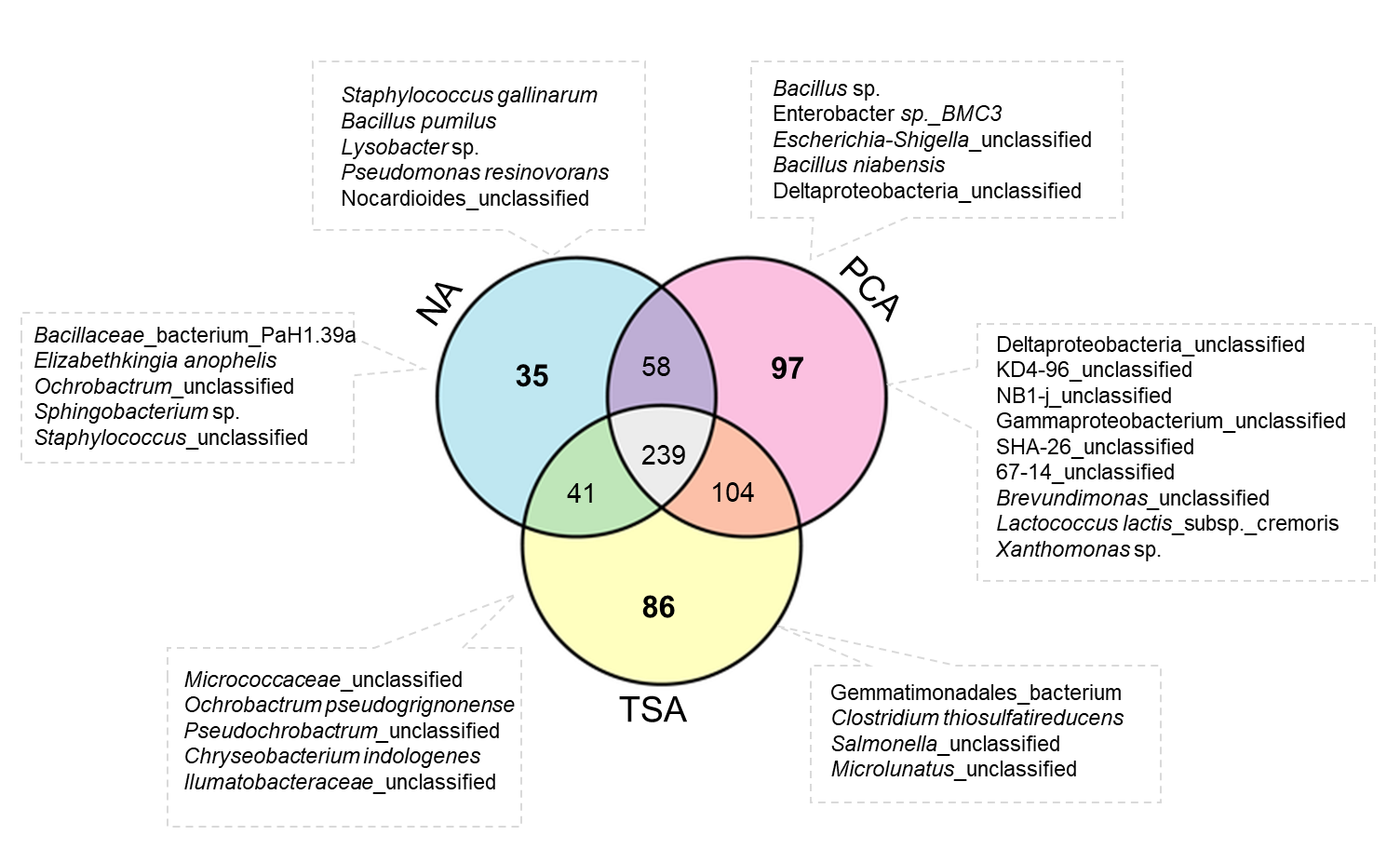

Supplement: Supplementary file 1 [file biology-10-00569-s001.zip › FigureS1.tif]
